# High Cell Density Perfusion Process of Quail Cells Producing Oncolytic rVSV‐NDV

**DOI:** 10.1002/elsc.70035

**Published:** 2025-07-14

**Authors:** Lennart Jacobtorweihe, Sven Göbel, Markus Wolschek, Jennifer Altomonte, Udo Reichl, Yvonne Genzel

**Affiliations:** ^1^ Bioprocess Engineering Max Planck Institute For Dynamics of Complex Technical Systems Magdeburg Germany; ^2^ Nuvonis Technologies GmbH Vienna Austria; ^3^ Department of Internal Medicine II, Klinikum Rechts Der Isar Technische Universität München Munich Germany; ^4^ Chair For Bioprocess Engineering Otto‐von‐Guericke‐University Magdeburg Magdeburg Germany

**Keywords:** oncolytic virus production, perfusion process, process intensification, tangential flow depth filtration

## Abstract

Oncolytic viruses as agents for the treatment of various types of cancer have demonstrated their potential in many clinical studies over the past decades. In particular, rVSV‐NDV (a recombinant vesicular stomatitis virus [VSV] construct with fusogenic Newcastle disease virus glycoproteins) shows promising preclinical results. This is due to its safety profile, immunostimulatory effects, and efficacy based on strong syncytia formation. Since virotherapy requires a high input of infectious viruses, efficient production processes are needed. Good manufacturing practice (GMP)‐compliant CCX.E10 cells have been previously reported as a high‐titer‐producing rVSV‐NDV candidate in batch mode. Here, semi‐perfusion was used to test quail‐originated CCX.E10 cells for rVSV‐NDV production at high cell densities and in different cell culture media. The best condition was transferred to a full perfusion process in a 3 L bioreactor using a tangential follow depth filtration (TFDF) device for cell retention. The integrated depth filter with a pore size of 2–5 µm allowed 99.9% cell retention at viable cell concentrations (VCCs) of up to 20.6 × 10^6^ cells/mL and continuous virus harvesting. With this setup, we were able to produce 1.33 × 10^9^ TCID_50_/mL infectious virus with a 5‐fold increase in space‐time yield (STY) compared to a batch process as a control.

*Practical application:* Despite significant progress in oncolytic virus development, early research primarily focuses on viral design and therapeutic potential, often overlooking production challenges until later stages. This gap hinders clinical translation, as manufacturing high oncolytic virus doses (up to 10¹¹ infectious particles per injection) remains a major bottleneck. Implementing GMP‐compliant cell substrates alongside perfusion cultures is essential to overcoming the low yields of traditional batch production. These advancements have far‐reaching implications for reducing costs, increasing dose availability, and accelerating the clinical adoption of this promising immunotherapy.

**Abbreviations**
CRD: cell retention device
CSPR: cell‐specific perfusion rate
CSVY: cell‐specific virus yield
DF: depth filtration
DYN: Dynamis AGT medium
GMP: good manufacturing practice
HCD: high cell density
hpi: hours postinfection
MOI: multiplicity of infection
NDV: Newcastle disease virus
NTU: nephelometric turbidity unit
rpm: rounds per minute
RV: reactor volumes
SCGM: suspension culture growth medium
STY: space time yield
TCID_50_
: tissue culture infectious dose 50
TFDF: tangential flow depth filtration
TFF: tangential flow filtration
TOI: time of infection
VCC: viable cell concentration
VSV: vesicular stomatitis virus
VVP: volumetric virus productivity
wv: working volume

## 1 Introduction

Oncolytic viruses provide an interesting approach for cancer therapy by mediating direct lysis of cancer cells together with an induction of a strong immune response [1]. Especially those viruses encoding a fusion protein, such as Newcastle disease virus (NDV), are promising due to their efficient viral spread [2]. Worldwide, there are currently four different oncolytic viral therapeutics approved by different authorities. From these four, Talimogene laherparepvec (T‐VEC), based on Herpes simplex 1 virus, is the only widely approved oncolytic virus [3]. Nevertheless, many other viruses are currently evaluated as oncolytic agents in clinical studies [4].

rVSV‐NDV is a hybrid construct based on the vesicular stomatitis virus (VSV) backbone with surface proteins (hemagglutinin, neuraminidase, and a mutated fusion protein) of the NDV. The fusion protein facilitates virus entry into the cell. Whereas the native NDV fusion protein needs to be cleaved by an avian protease, the mutated fusion protein is cleaved by endogenous proteases and causes enhanced syncytia formation. Compared to oncolytic viruses causing a classical cytopathic effect, syncytia formation primarily leads to increased virus spread to surrounding cells through cell‐cell fusion reactions [2]. rVSV‐NDV proved its potential in murine studies against hepatocellular carcinoma and melanoma, resulting in a prolonged overall survival [2, 5].

In our group, we have assessed various strategies for potential clinical‐grade production of oncolytic rVSV‐NDV with different cell lines, including BHK‐21, HEK293‐SF, AGE1.CR.pIX, and lastly CCX.E10. As a result, we achieved infectious virus titers of 7.5 × 10^9^ TCID_50_/mL with BHK‐21 cells at 20.6 × 10^6^ cells/mL in a perfusion process [6]. Considering regulatory requirements such as good manufacturing practice (GMP)‐compliance, safety, and productivity, we identified the suspension quail cell line CCX.E10 as an attractive candidate. However, so far cultivation of CCX.E10 cells at high cell density (HCD) in perfusion mode has not been described. In batch mode, CCX.E10 cells showed potential to produce high NDV titers [7] and successful production of rVSV‐NDV, achieving a virus titer of up to 4.2 × 10^8^ TCID_50_/mL [8]. Intensifying a batch process into perfusion mode can lead to an overall better process performance with increased infectious virus titer and overall yield, if the cell‐specific virus yield (CSVY) can be kept constant [9, 10]. Nonetheless, process intensification in perfusion mode shows some challenges when working with lytic viruses such as rVSV‐NDV or influenza [11]. Cell retention devices (CRD) are crucial for a successful perfusion process to enable operation at HCD. Membrane‐based CRDs should allow for high product passage for a direct virus harvest, while avoiding membrane blockage due to cell debris. Moreover, they should not damage the cells by shear stress and which decreases cell viability, nor should virus quality (infectious titer) be negatively influenced. For an industrially relevant process, those systems need to be scalable [9, 12]. Tangential flow depth filtration (TFDF) combines the abilities of tangential flow filtration (TFF) and depth filtration (DF) with a pore size of 2–5 µm. It is a platform to overcome the aforementioned challenges for CRDs, while offering lower shear stress for cell cultivation [13, 14]. Production of rVSV‐NDV leads to further challenges for CRDs due to the formation of large syncytia. Although with BHK‐21 cells, syncytia of up to 120 µm were reported, in our previous batch process with CCX.E10, no syncytia were observed [6, 8].

In this study, we evaluated the maximum viable cell concentration (VCC) that CCX.E10 cells can reach in different media by using semi‐perfusion strategies. Subsequently, rVSV‐NDV production is evaluated, verifying possible conditions for virus production at higher cell concentrations for these cells. Surprisingly, the best cell growth conditions did not lead to the best virus production. Therefore, optimal conditions for virus production were transferred into a lab‐scale bioreactor connected to a TFDF system, demonstrating for the first time the performance of CCX.E10 cells in perfusion mode at HCD to produce rVSV‐NDV. This allowed a similar yield to the previously described BHK‐21 process by reaching up to 1.33 × 10^9^ TCID_50_/mL at a CCX.E10 cell concentration of 20 × 10^6^ cells/mL.

## 2 Materials and Methods

### 2.1 Cell Culture, Media, and Virus Seed

CCX.E10 cells were cultivated in either Dynamis AGT medium (DYN) (Thermo Fisher Scientific) or suspension culture growth medium (SCGM) at 37°C, 5% CO_2_, and 185 rpm (rounds per minute) in 125 mL baffled shake flasks (Corning). SCGM medium is based on Freestyle 293 Expression medium (Thermo Fisher Scientific), which is further supplemented with growth factors. Cells were routinely passaged twice per week, seeding at 0.8 × 10^6^ cells/mL.

Infections were performed under the same cultivation parameters with varying VCCs. rVSV‐NDV virus seed was generated in CCX.E10 cells and concentrated by ultracentrifugation with sucrose gradients for purification. Multiplicities of infections (MOI) of 10^−4^ were used for infections.

Adherent AGE.CR.pIX cells (kindly provided by ProBioGen) were used for the TCID_50_ assay and passaged twice a week in T‐flasks (Corning) in DMEM/F12 (1:1) medium with 5% fetal calf serum.

### 2.2 Batch Cultivation

For shake flask infections (working volume [wv] of 50 mL), CCX.E10 cells were infected at a VCC of approximately 2.0 × 10^6^ cells/mL. At the time of infection (TOI), cells were diluted twofold with fresh medium. The infectious phase continued over 84 h postinfection (hpi), and samples for determining VCC, TCID_50_, and basic metabolites were taken every 12 h.

### 2.3 Semi‐Perfusion

During semi‐perfusion, CCX.E10 cells were cultivated in 125 mL baffled shake flasks. Manual media replacement to maintain perfusion rates was performed during the cell growth phase, as described elsewhere [15, 16, 17]. Therefore, during cell growth, the substrate‐related yield coefficients and glucose consumption rates were calculated with Equations (1) and (2). Postinfection, a fixed perfusion rate of 1.6 reactor volumes (RV) per day was used. Using DYN, the cell‐specific perfusion rate (CSPR) was set to 90 pL/(cell × day) and for SCGM to 73 pL/(cell × day). Therefore, cells were transferred to 50 mL Falcon tubes and centrifuged for 5 min at 300 × *g* medium was replaced according to Equation (4).

(1) $$Y_{X,S}=\frac{x\left(t_{n+1}\right)-x\left(t_{n}\right)}{c_{S}\left(t_{n}\right)-c_{S}\left(t_{n+1}\right)}\;$$

$Y_{X,S}$ represents the substrate‐related yield coefficient [cells/mmol], *x*(*t*_n_) cell concentration at time *n* [cells/mL], and *c*_S_(*t*_n_) substrate concentration at time point *n* [mmol/mL].

(2) $$q_{s}=\frac{\mu }{Y_{X,S}}\;$$

The glucose consumption rate $q_{s}$ [mmol/(cell × *h*)] and μ as specific growth rate [1/h].

(3) $$\mathrm{CSPR}\;=\frac{q_{s}}{c_{\mathrm{gm}}-c_{\mathrm{gb}}}\;$$

Here, CSPR represents cell‐specific perfusion rate [mL/ mmol/(cell × h)], $c_{\mathrm{gm}}$ glucose concentration in medium [mmoL/mL], and $c_{\mathrm{gb}}$ threshold for glucose concentration in the bioreactor [mmol/mL].

(4) $$V_{e}=\frac{X\cdot \left(e^{\mu \cdot \Delta t}-1\right)\cdot V_{w}\cdot \mathrm{CSPR}}{\mu }$$

Calculation of $V_{e}$, the exchange volume [mL], X the VCC [cells/mL], μ specific growth rate [1/h], Δt time between media changes [h], $V_{w}$ wv [mL], and CSPR [mL/(cell × h)].

### 2.4 Perfusion

CCX.E10 cells were cultivated in a 3 L DasGip system (Eppendorf) with an initial wv of 1.3 L SCGM, at 37°C and 180 rpm with a pitched blade impeller. Cells were seeded from shake flasks at 0.8 × 10^6^ cells/mL. Aeration with air was controlled with an L‐drilled hole sparger, with flow rates ranging between 3 and 9 L/h to obtain a DO level of at least 50%. The pH was set to 7.20 and controlled by sparging CO_2_ with a deadband of 0.05.

**FIGURE 1:**
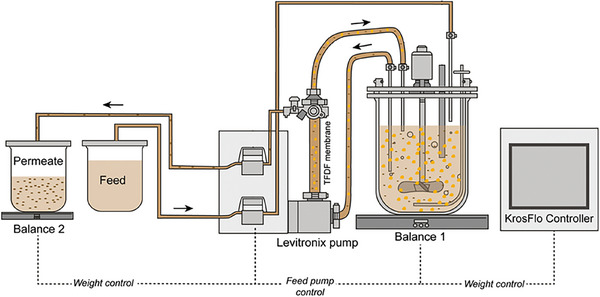
Scheme of the perfusion process using a TFDF device as CRD and perfusion rate controlled via weight. Cells are indicated in orange, viruses in brown circles. Figure adapted and taken from Göbel et al. [6].

The KrossFlow TFDF device (Repligen) was used for cell retention, with a polyethylene and polyethylene terephthalate 30 cm^2^ membrane and a pore size of 2–5 µm (Figure 1). Perfusion rates were controlled by weight (Figure 1) with the KrossFlo control system and manually set to 80 or 90 pL/(cell × day), after 72 h of batch growth. Every 12 h, the perfusion rate was adjusted according to the measured VCC, and a constant flow for the next 12 h was set. Two hours before infection, the perfusion rate was set to 10 mL/min to allow a full media exchange before infection. The KrossFlo controller was further used to monitor transmembrane pressure using its built‐in TFDF pressure sensors. At TOI, the bioreactor was infected at an MOI of 10^−4^, flow was paused for the first 2 hpi to prevent a seed virus dilution/wash‐out of freshly added seed virus. Afterward flow was fixed to 2 RV/d and reduced to 1.8 RV/d at 36 hpi. Post infection, samples for determination of turbidity, VCC, metabolites, TCID_50_, pH, and osmolality were taken every 12 h. Moreover, at each sample time, harvest bottles pre‐filled with 5% (v/v) sucrose to increase virus stability were exchanged and subsequently cooled to 4°C. The infectious virus titer of harvest bottles was determined to calculate the total produced infectious virus particles. A final harvest through the TFDF membrane was performed at 60 hpi to harvest not only the permeate, but also the remaining RV.

### 2.5 Analytics

VCCs and viabilities were determined using a ViCell XR (Coulter Beckmann). A Cedex Bio (Roche) was used to measure glucose, glutamine, glutamate, lactate, and ammonia concentrations. Samples were therefore centrifuged at 800 × *g* for 5 min, and virus‐containing supernatant was heat‐inactivated at 80°C for 3 min before analysis. Turbidity was measured from the bioreactor and permeate using a 2100Q turbidimeter (Hach). Virus titers were estimated by TCID_50_ as described earlier [18]. Briefly, adherent AGE.CR.pIX cells were seeded at 0.8 × 10^6^ cells/mL (100 µL/well) one day before infection in 96‐well plates. On the day of infection, samples were 10‐fold diluted up to 10^−9^ and pipetted in 8 replicates on 96‐well plates. After 72 h, plates were scanned for cytopathic effect (= positive well) under the microscope. The validation of the TCID_50_ assay resulted in an error of ± 0.3 log on a linear scale.

To evaluate the different experiments, CSVY, volumetric virus productivity (VVP), and space time yield (STY) were calculated.

(5) $$\mathrm{CSVY}\;=\frac{c_{\mathrm{vir},\max}}{X_{\max}}\;$$

CSVY, the CSVY [TCID_50_/cell] is calculated with *c*_vir,max_, maximum infectious virus concentration [TCID_50_/mL], and *X*_max_ the maximum VCC after infection [cells/mL].

(6) $$\mathrm{VVP}=\frac{\sum_{t_{0}}^{t_{n}}\left(TCID_{50\;H,t_{n}}\cdot V_{H,t_{n}}\right)}{\sum_{t_{0}}^{t_{n}}\left(V_{H,t_{n}}\right)\cdot t_{n\;}}\;$$

VVP represents volumetric virus productivity [TCID_50_/(mL × h)], TCID_50 H,tn_ infectious virus titer of the harvest at time *n* [TCID_50_/mL], and *V*_H,tn_ harvest volume at time *n* [mL].

(7) $$\mathrm{STY}=\frac{\sum_{t_{0}}^{t_{n}}\left(TCID_{50\;H,t_{n}}\cdot V_{H,t_{n}}\right)}{wv\cdot t}\;$$

STY represents the space time yield [TCID_50_/(RV × h)], wv as wv inside the STR [L], and *t* total time [h].

## 3 Results

Cell culture media have a major impact on cellular functions, such as growth and metabolism [19]. For the optimization of our rVSV‐NDV production in CCX.E10 cells, DYN and the standard medium SCGM were studied in more detail. To intensify the former batch process, the possibility of establishing a high‐yielding perfusion process was further investigated.

### 3.1 Comparison of Two Media for HCD in Semi‐Perfusion

**FIGURE 2:**
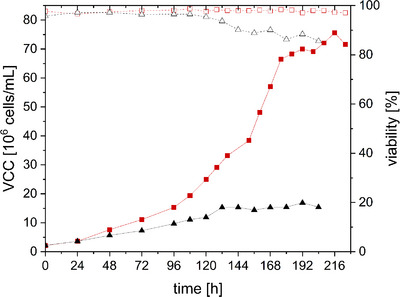
Growth of CCX.E10 cells in semi‐perfusion mode. Cultivation in DYN (■) or SCGM (▲) medium with CSPR of 90 and 73 pL/(cell × day) in 125 mL baffled shake flasks with 50 mL working volume. Viable cell concentration (VCC, full symbols), viability (empty symbols).

CCX.E10 cells are originally grown in SCGM, with reported doubling times of 26.6 h ± 2.6 h [8]. SCGM is a rather complex medium including multiple growth factors and putrescine. However, when trying to avoid the usage of some of the SCGM supplements, unfavorable growth characteristics and a decrease in virus titer were observed (data not shown). To further evaluate if both media could also support cell growth to higher cell concentrations (>10^7^ cells/mL), cells were cultivated in semi‐perfusion mode in shake flasks. Calculations for CSPR were based on previous experiments (data not shown) and were set to 90 pL/(cell × day) for DYN and 73 pL/(cell × day) for SCGM. Cell growth using SCGM was limited up to approximately 1.5 × 10^7^ cells/mL and a specific growth rate of 0.005 1/h (see Figure 2), although no limitation of glucose or glutamine or a critical accumulation of ammonia or lactate could be detected (see Supporting Information S1). Cell growth might have been limited by other metabolites, such as lipids or vitamins. DYN, however, supported cell growth up to 7.5 × 10^7^ cells/mL with a specific growth rate of 0.008 1/h, even with glucose concentrations being limited between 168 and 192 h (see Supporting Information S2).

**FIGURE 3:**
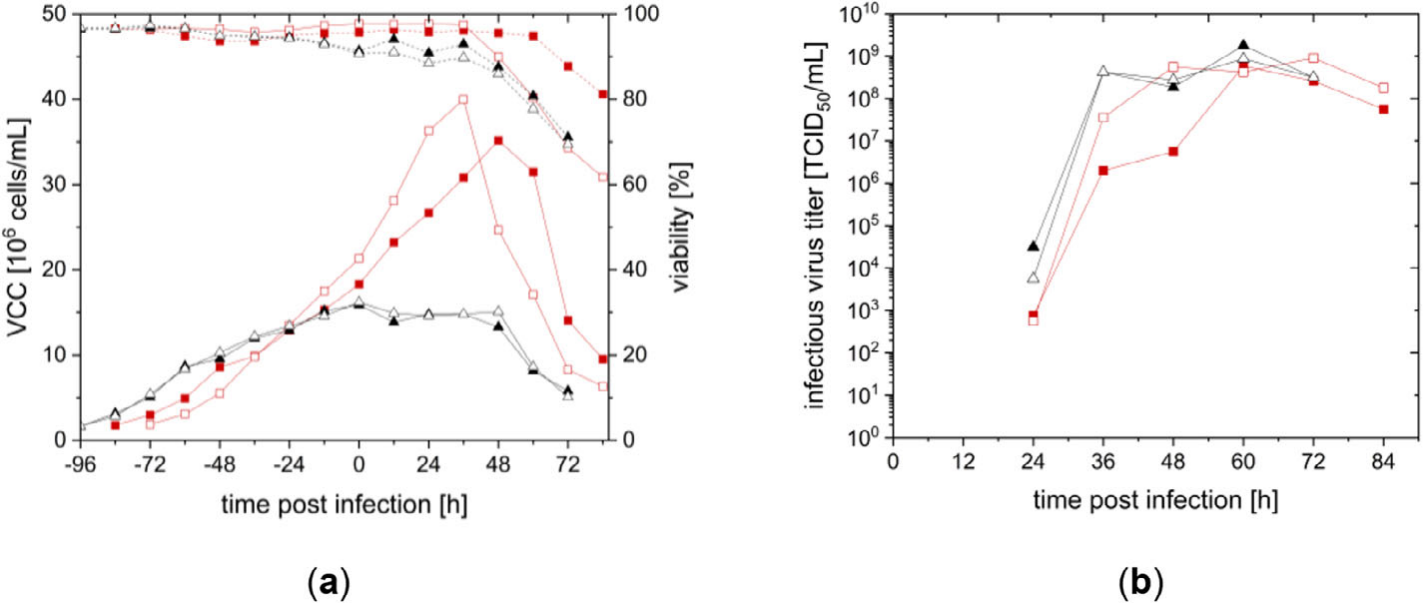
Growth and rVSV‐NDV production of CCX.E10 cells in shake flasks in semi‐perfusion mode, using DYN AGT (■/□), CSPR of 90 pL/(cell × day), or SCGM (▲/Δ) 73 pL/(cell × day). Infections were performed in duplicates at 1.5 × 10^7^ cells/mL and 2.0 × 10^7^ cells/mL at an MOI of 10^−4^. (a) Viable cell concentration (VCC) with full lines and viability in dashed lines (b) infectious virus titers determined with TCID_50_ assay.

In the next step, rVSV‐NDV production in semi‐perfusion in both media was evaluated. Cells were infected with rVSV‐NDV at an MOI of 10^−4^ at a VCC of 2 × 10^7^ (DYN) and 1.5 × 10^7^ cells/mL (SCGM). Optimal MOI and infection conditions for rVSV‐NDV in CCX.E10 cells have been determined before [8]. CCX.E10 cells with DYN continued growing to 3.5 × 10^7^ and 4.0 × 10^7^ cells/mL postinfection, followed by a reduced VCC and viability 36–48 hpi (Figure 3). First infectious titer could be determined 24 hpi. Maximum virus titers of the duplicates were achieved at 60 hpi with 5.86 × 10^8^ and 9.10 × 10^8^ TCID_50_/mL using DYN and 1.79 × 10^9^ and 8.75 × 10^8^ TCID_50_/mL with SCGM. The calculated CSVY for CCX.E10 cells in SCGM was thus 2.5–5 times higher than in DYN (see Table 2). In comparison to previous batch experiments, production in semi‐perfusion mode led to a 18‐fold higher infectious virus titer in DYN medium and 4‐fold higher titer in SCGM (see Table 2). A 3‐fold increase in VVP was observed using DYN, while for SCGM, no increase in VVP was observed (see Table 1).

**TABLE 1:** Comparison of rVSV‐NDV production in CCX.E10 cells for different media combinations in batch mode.

Medium in cell growth phase	DYN	SCGM	DYN +Suppl.	DYN	DYN: SCGM 1:4	DYN: SCGM 1:2	DYN: SCGM 4:1
**Medium in virus production phase**	DYN	SCGM	DYN +Suppl.	SCGM	DYN: SCGM 1:4	DYN: SCGM 1:2	DYN: SCGM 4:1
**Max. VCC** [10^6^ cells/mL]	3.5 ± 0.1	3.2 ± 0.2	4.8 ± 0.4	3.1 ± 0.1	4.7	4.8	4.1
**µ_max_ post infection** [1/h]	0.023 ± 0.004	0.022 ± 0.004	0.018 ± 0.002	0.017 ± 0.004	0.025	0.027	0.020
**Max. infectious virus titer** [10^8^ TCID_50_/mL]	0.4 ± 0.1	3.0 ± 1.7	0.5 ± 0.1	1.4 ± 0.6	1.8	1.0	1.3
**CSVY** [TCID_50_ /cell]	12.8 ± 5.7	98.0 ± 63.0	10.4 ± 1.3	45.9 ± 19.5	37.8	20.9	32.8

*Note:* The first four experiments were performed in duplicates (mean ± standard deviation).

Abbreviations: CSVY, cell‐specific virus yield; DYN, dynamis AGT; SCGM, Freestyle 293 + supplements; Suppl., supplements of SCGM; TCID_50_, tissue culture infectious dose 50; VCC, viable cell concentration.

### 3.2 Media Combinations for Further Increase in Virus Titers

**FIGURE 4:**
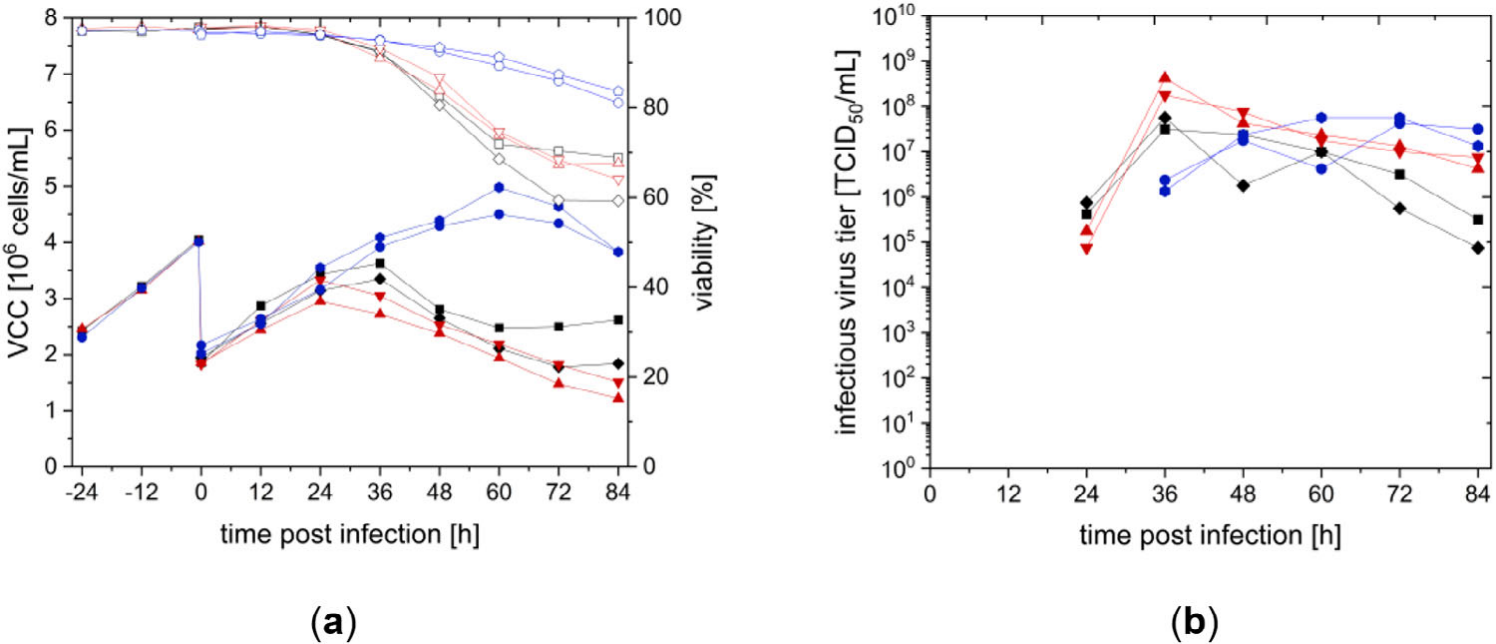
Growth and rVSV‐NDV production of CCX.E10 cells in batch mode (shake flasks) in different media: DYN (▲/µ), SCGM (■/◆), and DYN with SCGM growth factors (●/⬢). Infections were performed in duplicates at 2.0 × 10^6^ cells/mL with an MOI of 10^−4^ and a 2‐fold medium dilution step. (a) Viable cell concentration (VCC) with full lines and viability in dashed lines (b) infectious virus titers determined with TCID_50_ assay.

Follow‐up cultivations were performed to test whether combining the media or their supplements could achieve high cell densities while maintaining high CSVYs. Hence, in a first trial, supplements usually added to Freestyle293 medium to create SCGM were added to DYN to test their impact on the virus titer. Figure 4 shows the growth of CCX.E10 cells in batch mode with SCGM reaching their peak VCC of approximately 3.3 × 10^6^ cells/mL at 36 hpi, while in non‐supplemented DYN, cells grew up to 2.4 × 10^6^ cells/mL at 48 hpi. A 2‐fold higher VCC up to 4.9 × 10^6^ cells/mL was found using DYN with supplements, with a slowed loss in viability. However, infectious virus titer production showed a different pattern (Figure 4, right). Regarding CSVY (Table 1), the addition of supplements did not lead to increased virus productivity compared to the production in DYN only.

**FIGURE 5:**
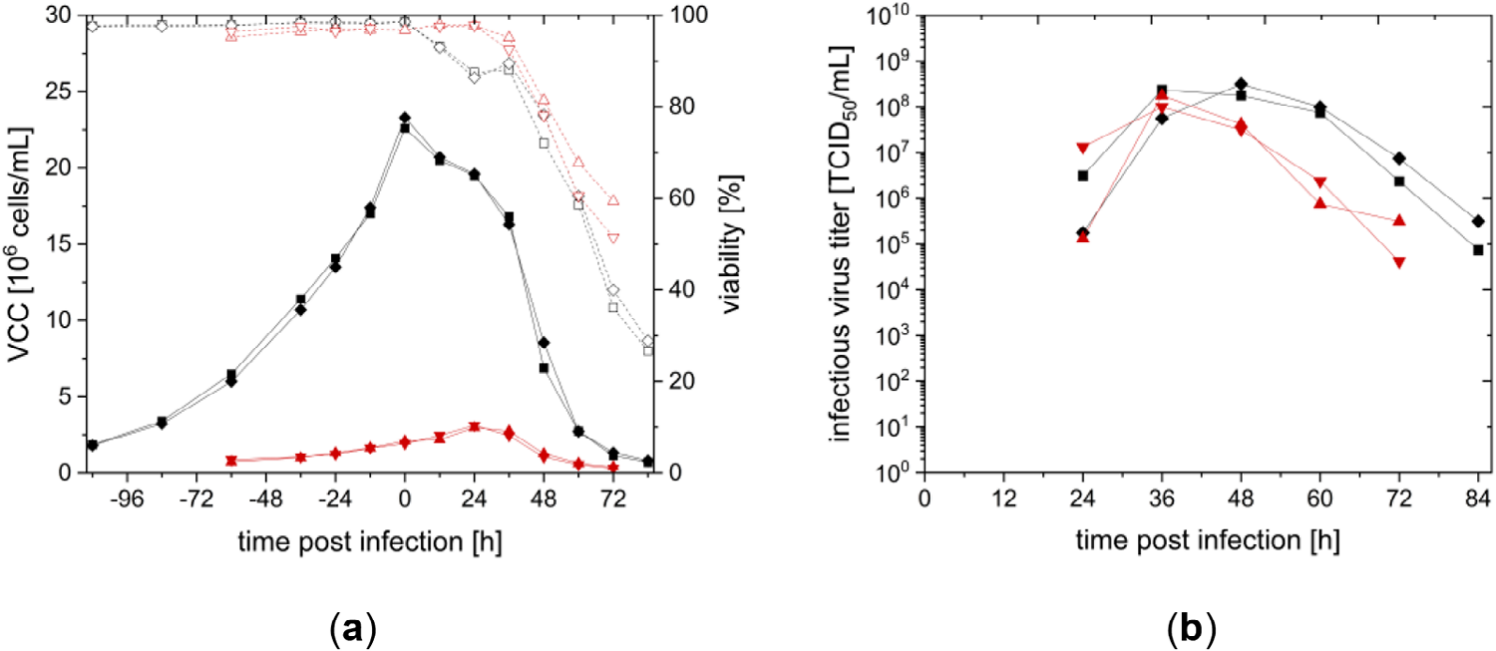
Growth and rVSV‐NDV production of CCX.E10 cells in semi‐perfusion (■/◆) and batch (▲/µ) mode. DYN medium was used during the cell growth phase and SCGM for the infection phase. Infections were performed in duplicates at an MOI of 10^−4^ at VCCs of 2 × 10^7^ cells/mL in semi‐perfusion or 2 × 10^6^ cells/mL in batch mode. (a) Viable cell concentration (VCC) with full lines and viability in dashed lines (b) infectious virus titers determined with TCID_50_ assay.

Since supplementation of DYN did not yield beneficial effects, a media change at TOI was tested. Cells were grown in batch mode and semi‐perfusion to compare low and higher cell densities. After growing CCX.E10 cells up to 2.1 × 10^6^ or 2.2 × 10^7^ cells/mL in DYN, for batch or semi‐perfusion mode, respectively, the medium was fully exchanged to SCGM and cells subsequently infected (Figure 5a). Compared to batch production in pure DYN or SCGM, the specific growth rate was reduced to 0.017 1/h (see Table 1). Peak titer in batch mode occurred at 36 hpi, while in semi‐perfusion highest titer was measured between 36 and 48 hpi (Figure 5b). In batch mode, CSVY increased 4‐fold to 45.9 TCID_50_/cell compared to batch cultivation using DYN medium (see Table 1). However, no increase in CSVY was found with a semi‐perfusion strategy. Moreover, rVSV‐NDV production with cell growth and virus production in SCGM was still superior.

**FIGURE 6:**
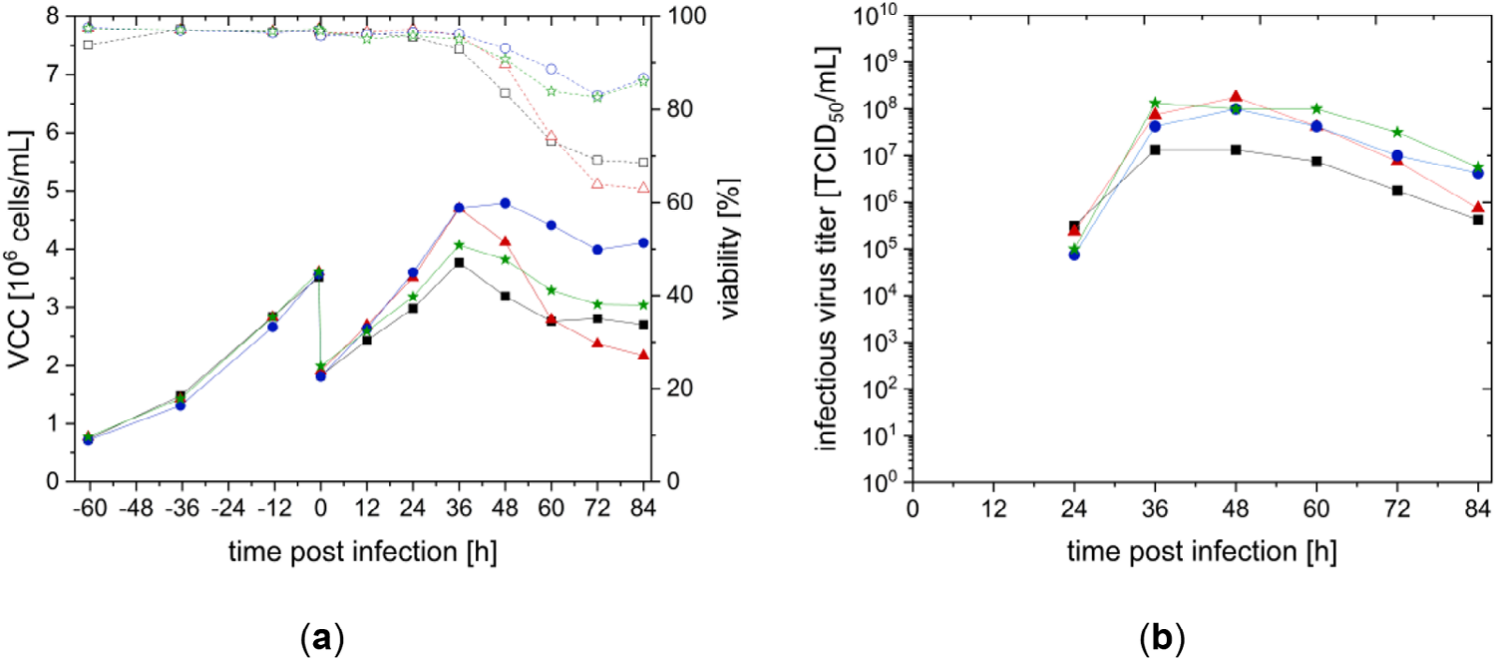
Batch cultivations with different DYN/SCGM ratios at an MOI of 10^−4^ with a 1:2 medium dilution step at TOI in shake flasks with 50 mL working volume. Tested ratios were: 100% DYN (■ DYN 100), 75% DYN and 25% SCGM (▲DYN 75), 50% DYN and 50% SCGM (● DYN 50), 25% DYN and 75% SCGM (★ DYN 25). (a) Viable cell concentration (VCC) with full lines and viability in dashed lines (b) infectious virus titers determined with TCID_50_ assay.

In a last experiment for media improvement, a blending strategy was used by mixing DYN and SCGM in four different ratios. Blended media were used for the cell growth and virus production phase, following a twofold medium dilution at TOI. The lowest infectious virus titer with 7 × 10^7^ TCID_50_/mL was obtained for 100% DYN (see Figure 6 and Table 1). An improvement in virus productivity, even when only adding 25% SCGM, was observed. Differences within the tested blending ratios are below the titration error. Since the virus titer only increased by 2.5‐ to 4.5‐fold, a production in SCGM still produced 1.6–3 times more rVSV‐NDV. Based on these findings, the further transfer into a stirred tank perfusion process with a TFDF membrane as CRD was carried out in SCGM.

### 3.3 Production of rVSV‐NDV in Perfusion Mode Using TFDF as CRD

TFDF combines the advantages of tangential flow systems and depth filters to operate at high cell densities. It enables cell retention with a direct harvest of virus particles and allows the integration of a first clarification step [6, 13, 20].

**FIGURE 7:**
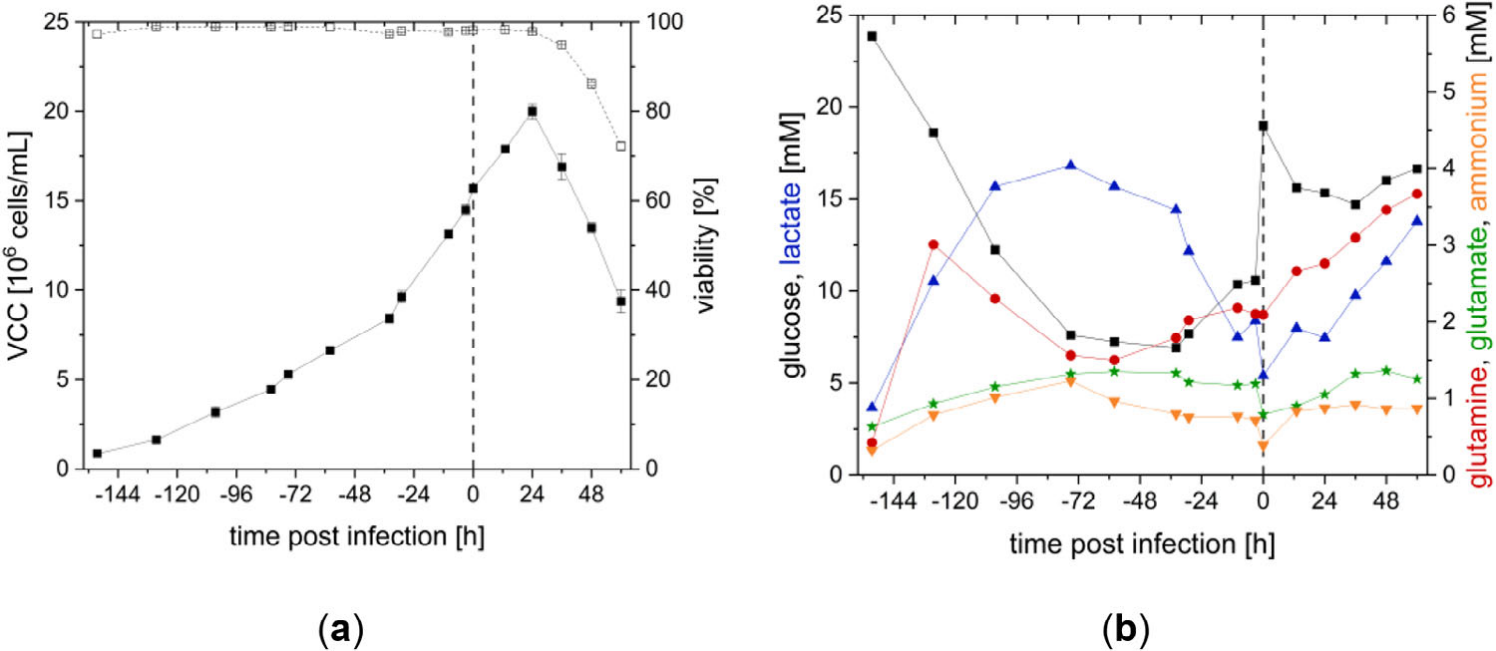
Cultivation of CCX.E10 cells in a 3 L STR (1.3 L wv with SCGM) in perfusion mode using a TFDF system and production of rVSV‐NDV (MOI of 10^−4^ at VCC of 1.6 × 10^7^ cells/mL). Perfusion started at ‐84 h to ensure optimal nutrient supply. (a) Viable cell concentration (VCC) with full lines and viability with dashed lines (b) main metabolites monitored, vertical dashed line implicates time of infection.

**FIGURE 8:**
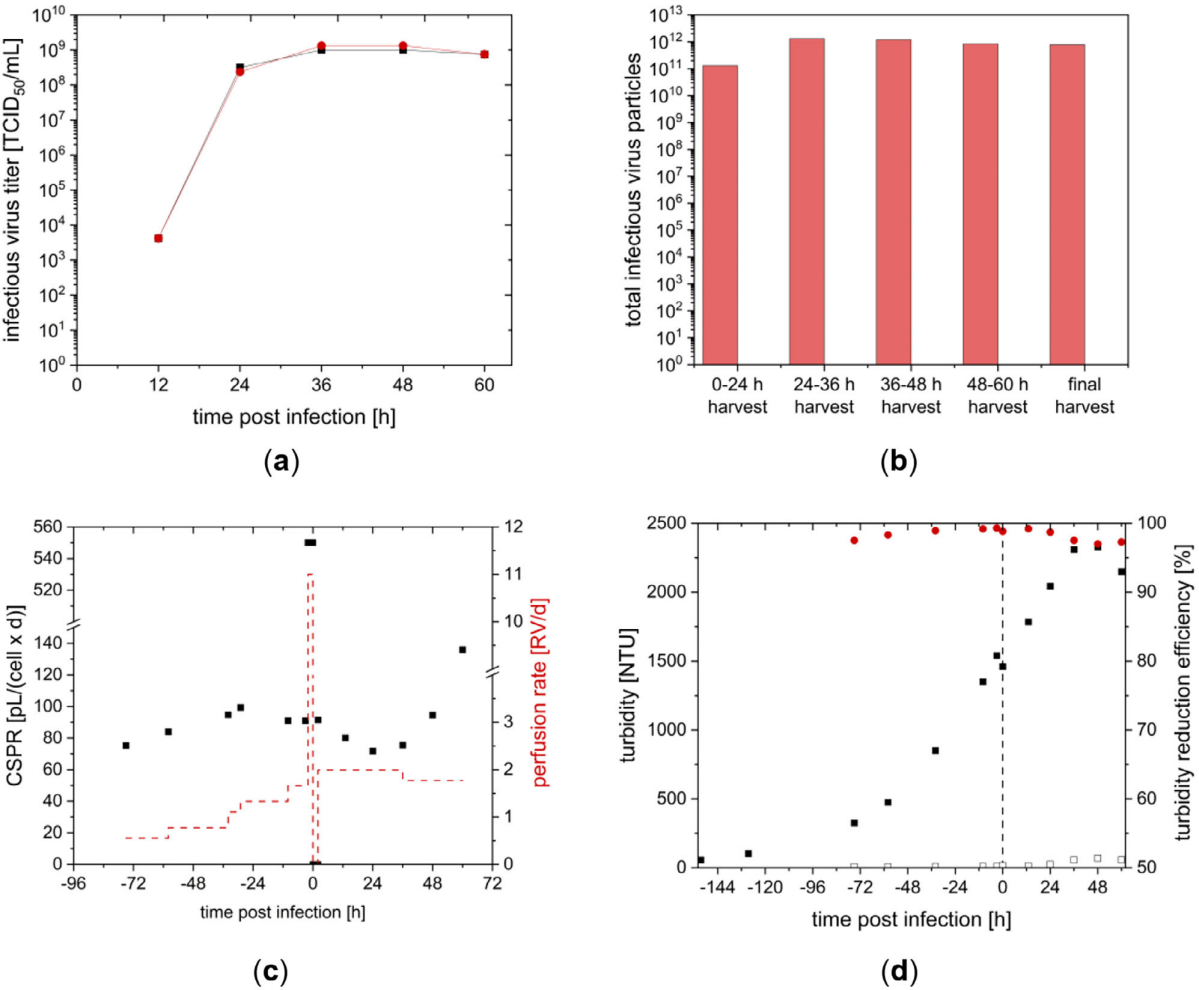
(a) Infectious virus titer during rVSV‐NDV production using CCX.E10 cells in perfusion mode (3 L STR coupled to a TFDF system) in the bioreactor (red) and permeate (black). (b) Total infectious virus particles harvested during the process and final harvest through the TFDF membrane. (c) Perfusion rates and CSPR during rVSV‐NDV production using CCX.E10 cells in perfusion mode. Pre‐infection a fixed CSPR of 80 pL/(cell × day) was used, which was increased to 90 pL/(cell × day) at −36 h. Postinfection, the perfusion rate was set to 2 RV/d and reduced to 1.8 RV/d at 36 h. (d) Turbidity was determined in the bioreactor (black full symbols) and the permeate (black empty symbols). Turbidity reduction efficiency is shown in red.

Cultivation of CCX.E10 cells was started in batch mode and switched to perfusion mode with a CSPR of 80 pL/(cell day) at −84 hpi. To maintain a base concentration of 8 mM glucose, the CSPR was raised to approximately 90 pL/(cell day) 36 h before infection (see Figures 7b and 8c). Surprisingly, perfusion mode enabled cell growth to a maximum VCC of up to 20 × 10^6^ cells/mL, clearly higher than what was seen in semi‐perfusion mode (see Figure 7a). Cell‐specific growth rate in perfusion mode was higher than in semi‐perfusion mode (0.019 1/h vs. 0.015 1/h). The chosen CSPR allowed to maintain glucose concentrations above 6 mM. Although ammonium concentration remained below a critical level of 2–3 mM, lactate concentration was comparably high between −108 h and −60 h before infection. Lactate concentrations of 20–30 mM can have a toxic effect due to changes in pH and osmolality [21]. As pH was controlled and cells kept exponentially growing, no effect of accumulated lactate was detected.

A characteristic drop in viability and VCC after infection was observed in this cultivation at 36 hpi [8, 22]. Comparable to the semi‐perfusion runs, a peak rVSV‐NDV titer of 1.3 × 10^9^ TCID_50_/mL was obtained at 36 hpi (see Figure 8a). As for the semi‐perfusion runs, infectious virus titers showed higher stability with almost constant values over 24 h. Based on semi‐perfusion data, the perfusion process was ended at 60 hpi once viability fell below 80%, to avoid a loss in infectious virus titer.

To evaluate the performance of the TFDF membrane with respect to a direct harvest of rVSV‐NDV, VCCs and infectious virus titers were measured from the bioreactor and permeate line. During the entire production process, cell retention efficiency was at 99.9%. Infectious virus titer measured in permeate and bioreactor was very similar (difference below the dilution error of the TCID_50_ assay), meaning no to little loss of produced virus in the TFDF membrane (see Figure 8a). Turbidity inside the bioreactor increased during the cultivation with increasing VCC, as shown in Figure 8d. After infection, VCC peaked at 24 h, whereas the highest turbidity was measured 48 hpi, probably related to cell lysis. The turbidity measured in the permeate reached a maximum of 70 nephelometric turbidity unit (NTU) 48 hpi, which was 33‐fold lower than in the bioreactor and comparable to the performance of depth filters [23]. Turbidity reduction and by that, retention of cell debris, did not lead to blockage of the TFDF membrane and, according to higher transmembrane pressure. Even after infection, the transmembrane pressure reported by the KrossFlow system stayed below a level of 0.12 psi.

In comparison to our previous data for a batch process [7], a 3‐fold increase in infectious virus titer was obtained in perfusion mode (Table 2). However, CSVY in perfusion mode was 45% lower and a 1.9‐fold decrease in VVP was observed. Nevertheless, STY increased by 5‐fold when compared to the STR batch process.

**TABLE 2:** Comparison of the production of rVSV‐NDV in CCX.E10 cells for different cultivation modes and media.

	DYN	SCGM	DYN	SCGM	SCGM [7]	SCGM
**Cultivation vessel**	SF	SF	SF	SF	STR	STR
**Process mode**	batch	batch	semi‐ perfusion	semi‐ perfusion	batch	perfusion
**Max. VCC** [10^6^ cells/mL]	3.5 ± 0.1	3.2 ± 0.2	37.6 ± 3.4	16.1 ± 0.2	4.2	20.3
**Max. infectious virus titer** [10^8^ TCID_50_/mL]	0.4 ± 0.1	3.0 ± 1.7	7.5 ± 2.3	13.3 ± 6.4	4.2	13.3
**CSVY** [TCID_50_ /cell]	12.8 ± 3.3	98.2 ± 63	19.7 ± 4.4	83.4 ± 41	100.0	65.5
**VVP** [10^10^ TCID_50_/(L × d)]	0.6 ± 0.2	4.2 ± 2.5	1.9 ± 1.1	2.9 ± 0.7	7.0	3.7
**STY** [10^10^ TCID_50_/d]	0.6 ± 0.2	4.2 ± 2.5	23.2 ± 13	32 ± 0.8	7.0	36.9

*Note:* The first four experiments were performed in duplicates (mean ± standard deviation). SCGM batch run results in STR were taken from Göbel et al. [7]. CSVY based on maximum VCC.

Abbreviations: CSVY, cell‐specific virus yield; SF, shake flask; VCC, viable cell concentration; VVP, volumetric virus productivity; STY, space time yield.

## 4 Discussion

Oncolytic virus therapy with rVSV‐NDV is an elegant approach to overcome current limitations in cancer therapy by specific infection of cancer cells and efficient cell‐to‐cell spread via syncytia formation, resulting in rapid immunogenic cell death [2]. However, intensified processes are needed to meet the virus dose requirements necessary for this therapy. For rVSV‐NDV, a dose of 5 × 10^10^ infectious viruses per injection is not unlikely. This study aims to intensify the existing batch process using CCX.E10 cells into perfusion mode, allowing production of the necessary yields for such dose requirements.

### 4.1 Comparison of Two Media for HCD in Semi‐perfusion

Semi‐perfusion as a scale‐down model in shake flasks allows a fast evaluation of perfusion rates and the overall ability to transfer a process into perfusion mode, even though process parameters, such as DO or pH, are not controlled [24]. Vázquez‐Ramirez et al. [17] tested shake‐flasks in semi‐perfusion mode as a scale‐down model for a full perfusion of AGE1.CR.pIX cells producing modified vaccinia Ankara virus. Applying the same perfusion rate for both systems, similar glucose and lactate concentrations were kept and further, pH varied in the same range of 7.2 ± 0.2 [17].

In previous batch processes, CCX.E10 cells grew up to 8.5 × 10^6^ cells/mL with SCGM. Here, the use of a semi‐perfusion method enabled 1.7‐fold higher maximum cell concentrations of up to 16 × 10^6^ cells/mL. Moreover, shifting to DYN medium allowed very high cell concentrations of up to 70 × 10^6^ cells/mL. When comparing glutamine, glutamate, ammonium, and lactate of SCGM and DYN medium, only minor differences were detected. Different glucose concentrations of 24 mM (SCGM) and 31 mM (DYN) seem not to be an explanation for the growth differences to high VCCs, since both cell lines show similar growth in batch mode, reaching 7.5 × 10^6^ cells/mL (DYN) and 8.8 × 10^6^ cells/mL (Supporting Information S3 and Göbel et al. [8]). Even though glucose concentrations above 8 mM were targeted in semi‐perfusion mode, CCX.E10 cells growth in SCGM was limited. We propose further components of the media formulation, for example, lipids or vitamins to be limiting at the CSPR of 73 pL/(cell × d). Using another avian cell line (EB66), Nikolay et al. showed that production processes for viral vaccines such as YFV and ZIKV, at very high VCCs of up to 160 × 10^6^ cells/mL, still result in efficient vaccine production without a decrease in CSVY. Compared to previous batch processes reported by Göbel et al., the growth rate of CCX.E10 cells was reduced by 35% to 0.017 1/h in semi‐perfusion mode and SCGM [8]. Slowed cell growth during semi‐perfusion was also observed by Bissinger et al. [15] when culturing MDCK cells in shake flasks. They proposed a possible effect from variations in medium temperature or pH, resulting in increased cell stress. Further, high fluctuation of metabolites due to “batch‐wise” exchange and no continuous exchange may cause a shift in the cellular metabolism.

CSVY differed strongly based on the medium used. Although DYN supported high cell concentrations, CSVY was 7‐fold lower compared to SCGM. Unlike production of recombinant proteins, viral infection often causes a strong metabolic shift inside the host cell, which was shown for several viruses, such as NDV [25]. Enveloped viruses can, for example, increase fatty acid and lipid synthesis, which might be used as additional membrane material [26]. Hence, a medium which supports cell growth might not necessarily be optimal for virus production as well.

Besides the difference based on the culture media used, the infectious virus titer increased for both experiments by changing the mode of production to semi‐perfusion. Infection of CCX.E10 at 15 and 20 × 10^6^ cells/mL led to an improvement of infectious virus titer by 18‐ and 4‐fold compared to a batch process when using SCGM or DYN, respectively. CSVYs in semi‐perfusion could be maintained at a comparable level to batch mode.

### 4.2 Media Combinations for Improved Virus Titers

An optimal medium would enable the production of high virus titer with low batch‐to‐batch variances [27]. Our data obtained from semi‐perfusion experiments implicate the efficiency of DYN medium for cell growth and SCGM for virus production. To combine both characteristics, different optimization experiments were performed. As commercial cell culture media are complex formulations and compositions are proprietary [28], optimization experiments are limited in complexity. In a first simple trial, supplements were added and cellular mechanisms treated as a black box [27]. Nonetheless, the addition of supplements to DYN only affected cell growth postinfection with up to 50% higher cell concentrations.

In a study by Shen et al., higher infectious virus titers were reached by the use of two media: one medium supporting cell growth and another for enhanced adenovirus production. By switching the medium at TOI, they achieved higher infectious virus titers [28]. Applying this strategy to DYN and SCGM improved CSVY 4‐fold compared to using DYN alone for both phases. However, using SCGM for both phases still resulted in 2‐fold higher CSVYs.

A frequently used medium optimization strategy, according to the literature, is medium blending. It is a simple and fast approach, where many conditions can be tested in a short time. As a drawback, it is limited in providing information on the exact components or mechanisms responsible for the impact of interest [29]. Medium blending with SCGM and DYN medium led to improved CSVYs compared to pure DYN medium. Variation of infectious virus titer between blending ratios is within the titration error. Even though slight improvements in CSVY were made, SCGM still showed better performance. Moreover, CCX.E10 cells in SCGM are GMP‐ready, while an adaptation of CCX.E10 cells to a new medium would likely require new characterization by the regulatory authorities, which might incur further expenses. Since cultivation and production in SCGM consistently demonstrated superior virus production, compared to all other variations in media composition tested, the transferred perfusion process in the DASGIP bioreactor system was carried out with SCGM.

### 4.3 Production of rVSV‐NDV in Perfusion Mode Using TFDF as CRD

Compared to our previous batch process, a 3‐fold higher infectious virus titer was produced, enabling high input doses. The CSVY in the final perfusion run was reduced by 45% compared to batch mode, and accordingly, a 1.9‐fold higher VVP was reached. However, a 5‐fold higher STY enables a smaller footprint of our current production process. Göbel et al. also showed an increase in rVSV‐GFP titer in HEK293‐SF cells using a TFDF as CRD with a 3.3‐fold lower VVP and 1.9‐fold higher STY [6]. This leaves room for improvement in further production runs by, for example, testing higher perfusion rates to maintain CSVY or infecting at lower VCCs. A comparison of batch, semi‐perfusion, and perfusion processes revealed an inverse relationship between maximum VCCs and CSVY, with higher VCCs resulting in lower CSVYs. This “cell‐density‐effect” is well documented in the literature and refers to the decline in cell productivity at higher cell densities [30]. Some perfusion processes showed the potential to overcome or, at least, reduce this effect [31, 32]. During our TFDF run, nutrient concentrations of glucose and glutamine were maintained at a sufficient level, while ammonium and lactate did not exceed reported inhibiting concentrations [21].

Based on the virus titers in the bioreactor and the permeate line, the use of TFDF as a CRD enabled continuous harvesting of rVSV‐NDV. Low transmembrane pressures indicated that no blockage of the used depth filter occurred. Other membrane‐based CRDs, such as hollow fibers, tend to retain produced viruses even though pore sizes should allow virus harvest, as reported for yellow fever virus (50 nm diameter) and pore sizes of 0.2 µm, or rVSV‐NDV and a pore size of 0.65 µm [6, 33]. Similar results using TFDF to enable virus harvest were shown before by Göbel et al., with recovery rates above 99% and transmembrane pressure below 0.3 psi [6, 34]. When produced in perfusion mode, rVSV‐NDV infection can cause large syncytia formation of up to 120 µm, as reported for BHK‐21 or HEK293SF [35]. However, for avian cell lines such as AGE.CR.pIX and here for CCX.E10, no syncytia were observed [35].

Harvesting through an integrated depth filter led to a 33‐fold reduction in overall turbidity. Thus, incorporating the TFDF membrane into the downstream process could eliminate one unit operation, streamlining production and purification. To further improve the process, a cooling tank for harvesting oncolytic viruses could be implemented to prevent potential virus degradation.

Using rVSV‐NDV in pre‐clinical studies, no dose‐limiting effects have been detected. Hence, safe doses above 10^10^ infectious viruses/dose can be assumed [35]. Besides an intensified upstream process, the subsequent downstream process is a further challenge in allowing the manufacture of a drug product with a sufficiently high infectious virus titer for use at effective doses in large numbers of patients. We have previously shown a four‐unit operation downstream process resulting in 64% overall recovery with 99% clearance of proteins and 97% clearance of DNA [23] for rVSV‐NDV produced in CCX.E10 cells. Assuming a similar recovery after downstream processing following the intensified production, the total infectious virus titer produced here in one perfusion run could yield 55 doses or 4.3 doses/L (with one dose of 5 × 10^10^ infectious viruses).

This work demonstrated that CCX.E10 cells could be successfully cultured at high cell densities while maintaining their CSVY in semi‐perfusion mode. The choice of the cell culture media was critical for cell growth and virus productivity. However, the media optimization strategies tested had no significant impact on increasing CSVY. CCX.E10 cells can be cultured in very high cell concentrations (>70 × 10^6^ cells/mL) in DYN medium and hence might be an interesting producer for other products as recombinant proteins. The process was successfully transferred from shake flasks to a 3 L DASGIP bioreactor using a TFDF device for cell retention, and a 5‐fold higher space time yield was achieved compared to the previously reported batch process. Overall, the described perfusion process demonstrated applicability to industrial production using a GMP‐compliant cell culture medium and cell line. Moreover, TFDF as a scalable CRD would allow for a scale‐up to a 2000 L bioreactor, potentially resulting in more than 8000 doses, assuming 4.3 doses per liter after downstream processing. Taken together, we present an intensified process that could be applied for the production of sufficient quantities of oncolytic rVSV‐NDV material for clinical trials.

## Author Contributions

Conceptualization, Methodology, and Investigation: L.J. and S.G. Writing – original draft: L.J. Writing – review and editing: L.J., S.G., M.R, U.R., J.A., and Y.G. Supervision: S.G., U.R., and Y.G. Project Administration: L.J. and S.G.

## Conflicts of Interest

J. Altomonte (WO 2017/198779) holds a patent for the development and use of rVSV‐NDV as an oncolytic therapy of cancer and is co‐founder of Fusix Biotech GmbH, which is developing the rVSV‐NDV technology for clinical use. M. Wolschek is an employee of Nuvonis Technologies, which owns the CCX.E10 cells. The other authors declare no conflicts of interest.

## Supporting information




**Supporting File 1:** elsc70035‐sup‐0001‐figuresS1‐S3.docx.

## Data Availability

Data are available in the article and the Supplementary Materials. Additional data are available on request from the authors.
