# Supplementary material for: High Cell Density Perfusion Process of Quail Cells Producing Oncolytic rVSV‐NDV
Source: Eng Life Sci. 2025 Jul 14;25(7):e70035. doi: 10.1002/elsc.70035 (PMC12256980; doi:10.1002/elsc.70035)
Supplement: Supplementary file 1 — Supporting File 1: elsc70035‐sup‐0001‐figuresS1‐S3.docx. [file ELSC-25-e70035-s001.docx]

**Supplementary material**





S1: Main supplement concentrations during CCX.E10 cultivation with SCGM in semi-perfusion mode to enable high cell concentrations in shake flasks.





S2: Main supplement concentrations during CCX.E10 cultivation with DYN in semi-perfusion mode to enable high cell concentrations in shake flasks.





S3: Growth of CCX.E10 cells in DYN and batch mode without infection (n=3).
